# Mechanical performance and dimensional stability of *Washingtonia*/Kenaf fibres-based epoxy hybrid composites

**DOI:** 10.1038/s41598-024-73300-3

**Published:** 2024-10-16

**Authors:** Naheed Saba, Sameer A. Awad, M. Jawaid, Mohamed Hashem, Hassan Fouad, Imran Uddin, Balbir Singh

**Affiliations:** 1 Laboratory of Biocomposite Technology, Institute of Tropical Forestry and Forest Products (INTROP), Universiti Putra Malaysia, 43400 Serdang, Malaysia; 2 Department of Medical Laboratories Techniques, College of Health and Technology, University of Al Maarif, Al Anbar, 31001 Iraq; 3 Chemical and Petroleum Engineering Department, College of Engineering, United Arab Emirates University (UAEU), PO Box 15551, Al Ain, UAE; 4 Department of Dental Health, College of Applied Medical Sciences, King Saud University, P.O. Box. 12372, Riyadh, Saudi Arabia; 5 Biomedical Engineering Department, Faculty of Engineering, Helwan University, Helwan, Egypt; 6 Centre for Global Health Research, Saveetha Institute of Medical and Technical Sciences (SIMATS), Chennai, Tamil Nadu India; 7 Department of Aeronautical and Automobile Engineering, Manipal Institute of Technology, Manipal Academy of Higher Education, Manipal, Karnataka 576104 India

**Keywords:** *Washingtonia filifera*, Natural fibres, Biocomposites, Tensile strength, Impact strength, Dimensional stability, Composites, Characterization and analytical techniques

## Abstract

In this study, Washingtonia fibres (AW) and Kenaf fibres (KF) were utilized as environmentally friendly fillers to improve the quality of the resin matrix. The mechanical, morphological, and physical properties of the WA/KF biocomposites were assessed throughout this research. The mechanical tests (tensile strength and moduli, elongation at break, flexural strength along with moduli, and the impact properties) were carried out. The hybrid biocomposites (3AW/7KF) exhibited the highest tensile strength (16.05 MPa) and modulus (4.6 GPa) among pure and other hybrid biocomposites. The impact strength and resistance of hybrid biocomposites (1AW/1KF and 7AW/3KF) showed the highest impact strength (1694 J/m^2^) while the 3AW/7KF hybrid biocomposite, the impact strength value was 1630 J/m^2^ (17.2 J/m). SEM images indicated good distribution and bonding of hybrid biocomposites. The investigation using morphological tests (Scanning Electron Microscopy (SEM)) displays the longitudinal roughness on the surface, which acts as a very significant function in the adhesion between the AW/KF fibres and the resin. Furthermore, the results of SEM confirm better bonding in the biocomposites, fibre fracture, pull-out, fibre shearing, and tearing in the pure and hybrid composites. From the water absorption test, it was observed that, when increasing the immersion time of biocomposites, the WA percentage of KF biocomposite significantly increased (37%) compared to other biocomposites. However, the hybrid and pure biocomposites exhibited more resistance to increase the WA percentage after increasing the immersion times, compared to other biocomposites. Furthermore, the thickness swelling (TS) of hybrid biocomposites increased compared to pure biocomposites. The biocomposite sample (3AW/7KF) was thicker on the 7th day exhibiting the greatest increases in thickness swelling (4.98%) while the hybrid biocomposite exhibited greater WA value compared to other correspondence samples. Finally, the KF and AW hybrid blends can be appropriate for several applications, for example, textiles, machinery part production industries, medicine, and automobiles, and construction, specifically buildings, bridges, and structures such as boat hulls, swimming pool panels, racing car bodies, shower stalls, bathtubs, storage tanks. Overall, the findings exhibit that the hybridisation of natural fibres (KF/AW) is a sustainable approach for obtaining biocomposites with advanced mechanical and thermal performance. Hence, they could be used in numerous specific applications, including automobile panels, structural products, sporting goods and furniture tools.

## Introduction

The reinforcements of natural fibres have involved extensive consideration in the industry of composite, due to their particular mechanical characterization and environmental benefits^1–4^. Natural fibres are available in abundance, accessible, biodegradable, as well as cost-effective.

They have significantly replaced the use of synthetic fibres, for example, glass and carbon^1–3,5–7^. Though natural fibres have the potential to be used as a substitute for synthetic fibres for a variety of purposes, their application however requires qualification, due to their mechanical properties, which differ from those of reinforcements commonly used in industry^3,5,6^. Furthermore, for many manufacturing applications and service, hybrid biocomposites are strengthened with natural fibres to decrease the usage of synthetic materials^8^.

Epoxies grant low viscosity prior to cure, which supports in wetting of fibre and saturation. This prevents minimising air voids and maximises adhesion between the polymer matrix and the strengthening fibre. Together, these factors help determine the strength of the overall epoxy composite^9^.

Currently, the use of natural fibres as strengthening materials in polymer composites has piqued the interest of several researchers around the world, owing to concerns about climate change, greenhouse gas emissions, deforestation, non-biodegradable waste, and air and water pollution, all of which are major concerns for humanity^10,11^. In addition, the construction of hybrid biocomposite for fibre-strengthened polymers are due to their potential benefits involving low/weighing applications^12^. The request for biodegradable materials produced from natural fibre-reinforced polymers in manufacturing areas has been increasingly growing due to mechanical mechanisms that require lightweight samples, greater strength, and stiffness, besides fatigue resistance, in addition, to ease of production and lower cost than synthetic fibres^13^. Moreover, researchers have ranted that hybridisation is a potential key to the restrictions of natural fibres as that would decrease the application of synthetic fibres and their associated environmental effects^14,15^. Another related research paper investigated the mechanical properties and absorption behaviour of ramie and pineapple leaf fibre (PALF)–reinforced epoxy hybrid composites, showing significant improvements in tensile, flexural, and impact strengths in addition to improving the water absorption by up to 12%^16,17^. In another study, hybrid biocomposites (ramie/flax natural fibres) were assessed using various techniques. It was found that the hybrid biocomposite exhibited improvements in mechanical properties. Furthermore, the Impact strength of the hybrid combinations increased from 10.23 to 15.97 kJ/m^2^ while the maximum tensile strength of the hybrid biocomposite was 33.46 MPa^18^. Another study showed that the incorporation of hybrid natural fillers into the epoxy matrix, resulted in significant improvement in their mechanical properties^16,17^.

In this study, Washingtonia filifera fibre is used as a strengthening component for the polymer matrix, resulting in an ecological advantage. When compared to the more well-studied jute^19^, sisal^20^, and flax fibres^21^, this fibre exhibits suitable mechanical properties. Furthermore, *Washingtonia filifera (AW)* is a palm that provides the most accessible and cost-effective source of natural fibres when compared to other sources, which include a diverse range of fibrous goods, such as the trunk, petiole, and leaves crumpled into a single fan^22^. Enhancing a biocomposite with WF fibres enables the valuable utilisation of a local natural resource^23^. Kenaf fibre, on the other hand, is made from the best plant stems of *the Hibiscus, Malvaceae* family*, and Cannabinus* genus. Kenaf fibres require less water to cultivate. They have the advantage of being renewable, low-cost, light, and non-hazardous. As a result, they can serve as an excellent polymeric material strengthening agent^24,25^. Kenaf fibre has a relatively high specific strength and stiffness, making it appropriate for use as a strengthening ingredient in resins to create an effective structural composite. It has previously been approved for use in vehicle components due to its lightweight and excellent mechanical properties^26^. Despite the fact that kenaf fibres have documented deficiencies typical of natural lignocellulosic fibres, it is a somewhat encouraging factor in the strengthening of polymeric composites. The cellulose composition (31%–72%) and microfibrillar angle (9°–15°) alter the mechanical characterisation of the kenaf fibres, yielding considerably higher tensile strength^27^. Fundamentally, it was discovered that polypropylene reinforced with kenaf fibres exhibits superior tensile and flexural properties when compared to other polypropylene-reinforced fibres, such as hemp, glass, and sisal fibres, which makes its application as a characterised and effective material for automotive structures in the industry^28^.

Essentially, composites strengthened with kenaf have utilised differentiated polymers comprising polypropylene^29–32^, high-density polyethene^33,34^, polystyrene^35^, polylactic acid^36,37^, natural rubber^38^, and epoxy resins^39^ amongst others. Specifically, resin matrix strengthened with kenaf fibres was examined for various functions before.

Park et al.^39^ assessed the micromechanical characterisations of biocomposites (resin/KF) as well as kenaf fibre’s wettability utilising emission of non-harmful acoustic. Xue et al.^40^ showed significantly enhanced mechanical characterisations, for example, elasticity modulus, and tensile strength for biocomposites strengthened by kenaf fibres.

Regardless of these substantial findings, no systematic study has been performed, to our knowledge, on the mixed chemical and thermal performance of kenaf fibres enhanced biocomposites to produce realistic constraints for feasible manufacturing requirements. Incomplete data on resin composites may be noticed comparably due to the higher cost involved when compared to other resins. Nonetheless, for high-value applications, resin provides excellent adherence to strengthening fibres and improved mechanical characterisation due to low moisture content, decreased shrinkage after curing, and ease of processing at ambient temperature.

In this study, natural fibres enhanced hybrid biocomposites were made from different fibres to investigate the impact of hybridisation on mechanical, physical, and morphological properties when compared to pure fibres. It is assumed that the hybridization of *Washingtonia Leaf Stalk Fibres (AW)* with Kenaf fibres (KF) will improve interfacial bonding, hence improving tensile and flexural properties, and the resin will have high dimensional stability. Mechanical (tensile, flexural, and impact) characterisation, physical (water absorption and thickness swelling), and morphological (SEM) investigations were performed on the biocomposite samples. This study introduces a method inspired by the fabrication of hybrid fiber biocomposites (AW/KF) to enhance their mechanical, physical, and morphological properties. Additionally, this research aims to advance the production of biocomposites through the hybridization of different natural fibers (NF), making them more suitable for industrial and engineering applications, including automotive, roofing, and other outdoor industrial uses.

## Materials and preparations

### Materials

In this work, a liquid resin type (D.E.R.™ 331™) representing the chemical reaction of manufacturing of Epichlorohydrin in addition to Bisphenol A, supplied by (Dow, Chemical Pacific-Singapore), as well as a corresponding hardener (Jointmine: 905-3 S) produced by (Epochemie Int. Pte. Ltd., Taiwan), was mixed in a mass (2:1) proportion. Table 1 shows the properties of *Washingtonia Leaf Stalk Fibres (AW)* and Kenaf Fibres (KF) used here.

**Table 1:** The mechanical and physical properties of natural fibres used in this study.

Property	Washingtonia Leaf Stalk Fibres (AW)	Kenaf Fibres (KF)	References
Tensile strength (MPa)	134.22	282.60	Asim et al.^62^
Tensile modulus (GPa)	2.17	7.13	Asim et al.^62^
Density (g/cm^3^)	1.07–1.12	1.26	Asim et al.^62^
Moisture absorption (%)	-	12–20	Arjmandi et al.^63^
Fiber length (μm)	252	83.5	Asim et al.^62^
Cellulose content (%)	-	66.89	Asim et al.^62^
Hemicellulose content (%)	-	14.98	Asim et al.^62^
Lignin content (%)	-	6.85	Asim et al.^62^

### Biocomposite fabrication

**Fig. 1:**
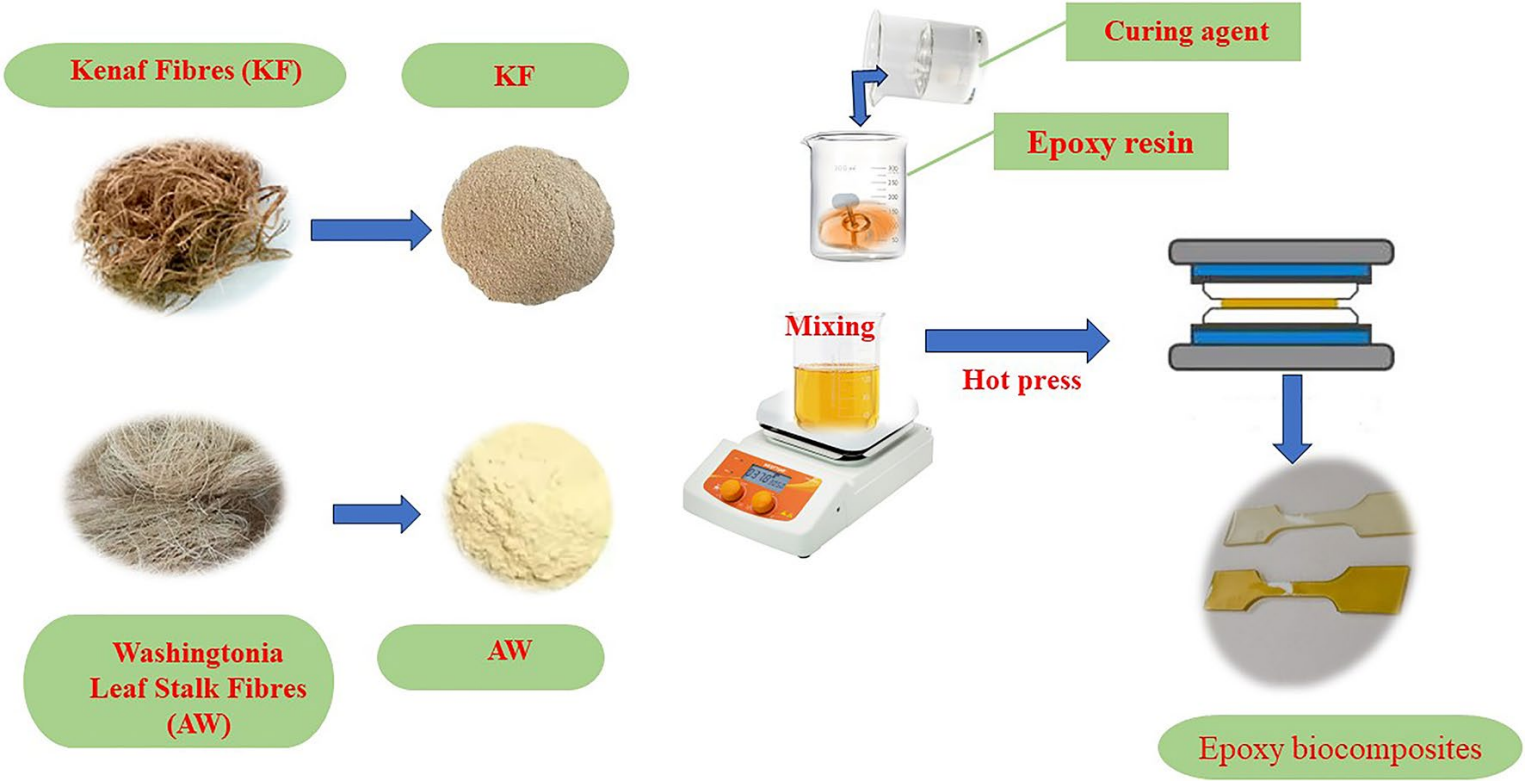
A scheme shows the preparation of biocomposites.

The WA and KF fibres were reinforced in a bio-resin matrix, with the fibre content maintained at 40% by weight and a density of 1.2 g/cm^3^. After being dried in an oven at 60 °C for up to 24 h, the specimens were stored in a laboratory oven at a temperature range of 70–80 °C for 4–5 days. The dried specimens were then ground into fine dust with an average particle size of 2 μm using a crusher. The resin and fibres were mechanically mixed and then poured into a stainless-steel mould (155 mm × 155 mm × 4 mm) coated with a thin layer of silicone spray. The mixture in the mould was cured in a hot press at 115 °C for 10 min, followed by cooling in a cold press for 5 min. The samples were then de-moulded and used to test various mechanical properties of the reinforced fibres, as shown in Fig. 1. For comparison, an epoxy sample was synthesized without reinforcement. Table 2 presents the formulation and nomenclature of the biocomposites^41^.

**Table 2:** Formulation and nomenclature of biocomposites^41^.

Hybrid samples	The total fibre percentage in the composite is 50%		Epoxy resin (Wt. % of composite)
	*AW* (Wt.% of Fibre)	Kenaf Fibre (KF) (Wt.% of Fibre)	
50%AW	50	0	50
50%KF	0	50	50
3AW/7KF	30	70	50
1AW/1KF	50	50	50
7AW/3KF	70	30	50

## Characterization technique

### Tensile testing

Following the standard ASTM. D: 638-03, the tensile test was achieved by utilising (Bluehill-INSTRON: 5567, UTM, USA) equipment together by a grip force of around (30 kN). The testing was achieved at an ambient temperature of around 23 ± 3 °C and relative humidity^42^ of approximately (50 ± 5%) performing a tensile measurement for the specimen (165 mm × 20 mm × 5 mm).

### Flexural testing

The tests of three-point flexural for composites were performed, utilizing a universal testing machine: Bluehill INSTRON5567 (Shakopee, USA) at 23 ± 5 °C and relative humidity (50 ± 10) % as per ASTM D 790-03. The rectangular specimen with a cross-section is located on two supports and is filled halfway between the anchors employing a loading nose. The flexural modulus was determined using the initial section slope for deflection of the load curve.

### Impact test

Notched Izod impact test samples were prepared with the dimensions (70 mm × 15 mm × 5 mm) and measured/tested utilising (Gotech GT-7045-MD, Taiwan) following the standard ASTM D-256. The notch angle was kept at 45°, and the diameter was 2.6 mm. Each sample has undergone up to five replications.

### Scanning *electron* microscopy (SEM)

The micrographs of the surface morphology for fractured biocomposite specimens were also taken using a Scanning Electron Microscope setup; EM-30AX, m COXEM- Daejeon-Korea; to analyse specimen failure. The images were captured at a magnification rate of 1000 times the original, for a 10 μm sample at a power supply of 20 kV. Samples on the SEM holder were then focused on, determining the fracture properties like matrix cracking, delamination, adhesion etc.

### Dimensional stability

The ASTM D 570–988 standard was used to analyse the fabricated WA and TS samples of these biocomposites with dimensions of (20 mm × 20 mm × 5 mm). Before immersion in distilled water, the initial mass and thickness of the tested samples were examined and documented. Before immersing in distilled water, the mass and thickness of samples were examined every 24 h for a week.

The WA and TS values of biocomposites were estimated based on the following equations (Eq. 1 and Eq. 2), respectively^43^

1 $$Water Absorption \left(WA\right)=\frac{{W}_{1}-{W}_{0}}{{W}_{0}}\times 100$$

where *W*_1_ is the weight of samples after the immersion while *W*_0_ is the sample’s weight before the immersion

2 $$Thickness Swelling \left(TS\right)=\frac{{T}_{1}-{T}_{0}}{{T}_{0}}\times 100$$

*T*_1_ represents the thickness after the soaking while the *T*_o_ represents the thickness before soaking.

## Results and discussion

### Tensile testing

**Fig. 2:**
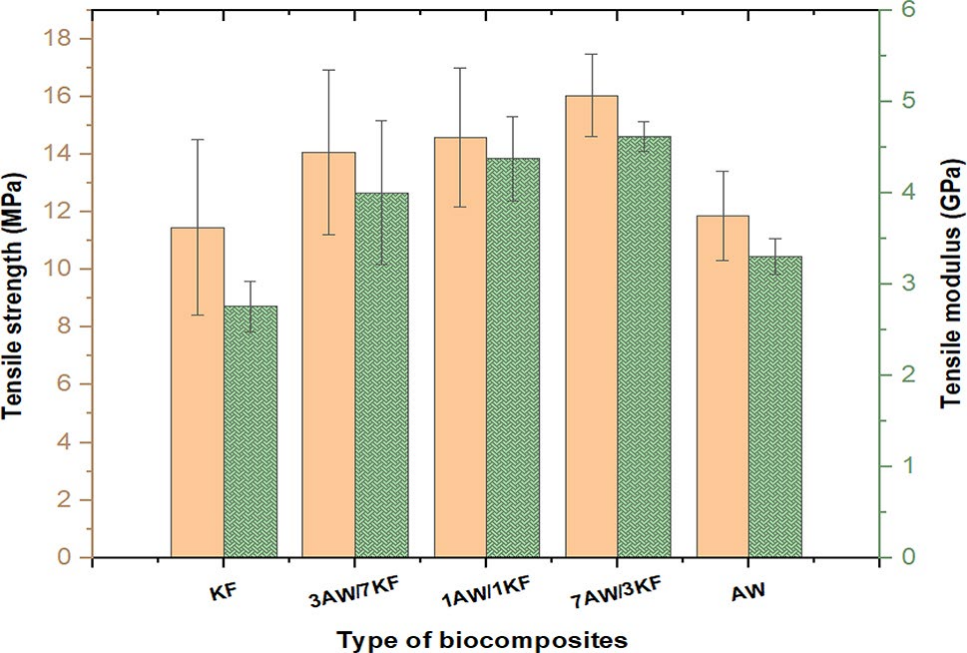
Tensile strength and modulus values of various pure and sample biocomposites.

**Fig. 3:**
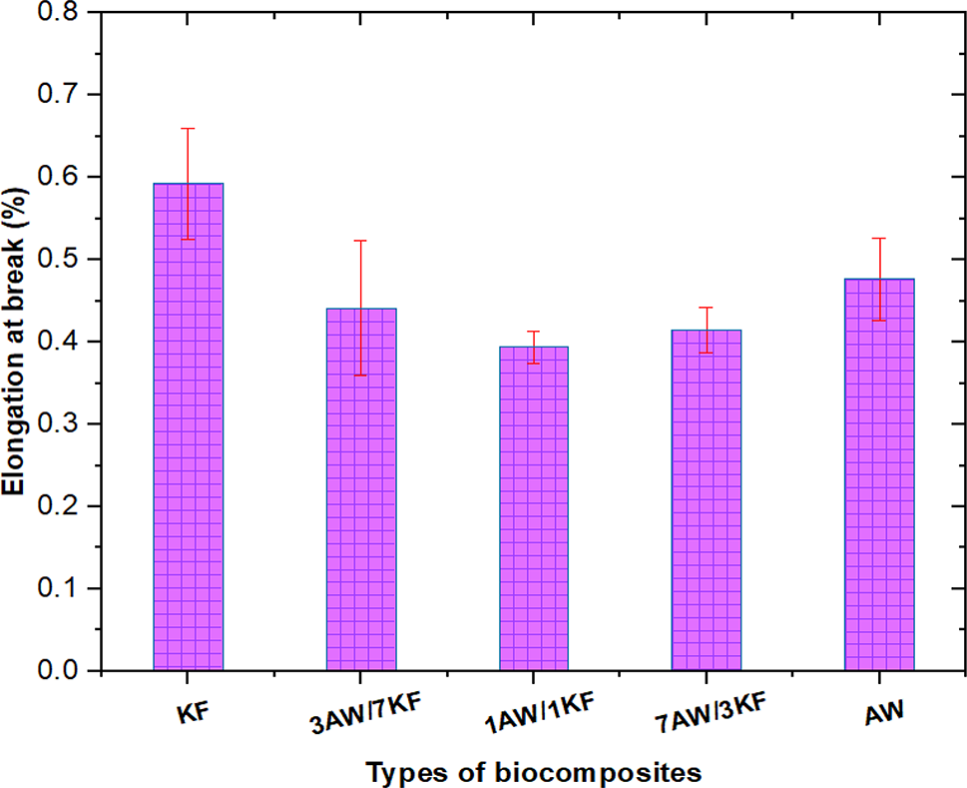
Elongation at break values of pure and sample biocomposites.

Various tensile characterizations, such as tensile strength and tensile modulus, as well as elongation at break, are measured as a function of the load rate of natural fibres in this testing, as shown in Figs. 2 and 3. Tensile strength, tensile modulus for pure biocomposites (KF and AW), and elongation at break of KF biocomposite are 11.48 MPa, 2.75GPa, and 0.59%, respectively, as shown in Fig. 2. It appears from the results that, for biocomposite (AW), the tensile strength and modulus exhibited a slight increase of 11.87 MPa and 3.3GPa respectively, while the elongation at break was slightly reduced to around 0.48%, as shown in Fig. 2. On the other hand, for the hybrid biocomposites, the tensile strength of 7AW/3KF biocomposite increased to 16.05 MPa compared to the corresponding samples 1AW/1KF and 3AW/7KF which are 14.59 MPa and 14.08 MPa, respectively. Figure 2 also revealed that the tensile strength and modulus of hybrid samples improved significantly compared to pure biocomposites. This may be due to that the incorporation of hybrid natural fibres shows better tensile strength than pure natural fibre composites because the hybrid fibres have more hydrophobic fibre, which enhances compatibility between fibre and matrix, hence increasing strength, stiffness, and interfacial adhesion of biocomposites^42^.

In comparison to this work, Chandrasekar et al. investigated the improvements of flexural, impact and dynamic mechanical characterisations of the Pineapple leaf fibres (PALF) and *Washingtonia trunk fibres (AW)* based bio-phenolic hybrid composites were examined. They revealed that 1AW/1P hybrid composites exhibited 25% and 12% improvements in flexural strength and modulus compared to the AW composites. 7AW/ 3P biocomposites showed a twofold increase in impact strength than AW composites^44,45^.

Khan et al.^46^ analysed the tests of tensile strength of hybrid kenaf fibre/ Jute Fibre (KF/JF) biocomposites. They found that the maximum tensile strength observed in the hybrid sample (KF/JF), was about 88.08 MPa compared to the pure biocomposites^47^. A number of researchers have investigated the mechanical characterisations of hybrid natural biocomposites. For example, Khan et al. investigated the impact of woven kenaf (K) and jute (J) fibre stacking sequences on the mechanical characterisations of epoxy biocomposites. They found that that the greatest improvement in the tensile and flexural characterisations was in the K/J/K bio composite. Moreover, the values of tensile strength for the K/J/K biocomposites had the greatest tensile strength of 43.21 MPa contrasted to the J/K/J biocomposite, which had 40.66 MPa. The strong tensile strength of the woven kenaf fibers maybe due to the outside skin indicates they are capable to manage with the tensile stress, whereas the jute core is pressure-bearing and constantly spread in hybrid biocomposites. In related previous work performed by Chandrasekar et al., studies the mechanical properties of hybrid biocomposites strengthened with Washightonia trunk fibres (GW)/Pineapple fibre (PALF) in the bio-phenolic matrix. They observed that hybrid biocomposite (7P3G) exhibited greater tensile strength and stiffness, which of 21.92 MPa and 4.36 GPa, respectively. This indicates that the hybrid fibres represent load carriers, transferring stress from the resin matrix to the fibres, producing a uniform and efficient stress distribution, which improves the mechanical characterisations of biocomposites^44,45^.

Building on similar research, it was demonstrated that the inclusion of porcelain fillings significantly enhances the composite’s tensile strength, flexural strength, and impact resistance, as evidenced by mechanical testing^48^. Related work showed that the hybridisation of Napier grass fibre/porcelain particles, improved the mechanical performance, for example, the tensile strength was increased by 25%^49^. In comparison with another study, it showed that the hybrid biocomposites (areca fibre (AF)/ glass fibres exhibited the greatest value of tensile strength (24 MPa) while the impact strength was improved by 34%^50^. Another related study showed that the biocomposite hyacinth ash particles included with eggshell filler enhanced the mechanical strength and hardness^51^. Arivendan et al. showed that the addition of 30% of the hyacinth fibres in epoxy composite improved the physical and mechanical properties^52^.

### SEM morphology study

**Fig. 4:**
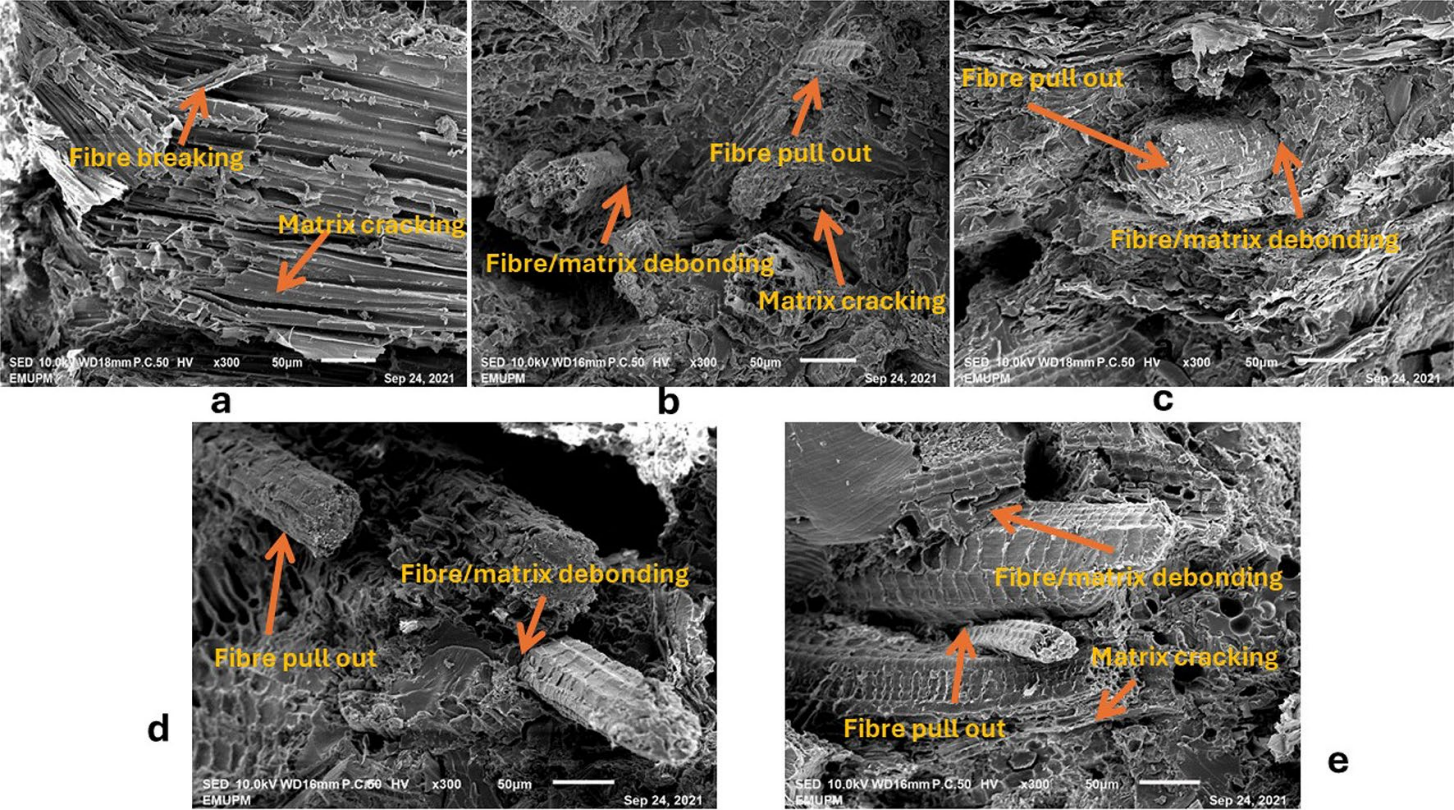
SEM micrographs of tensile fracture surface of fibres in biocomposites (**a**) KF (**b**) AW, (**c**) 3AW/7KF, (**d**) 1AW/1KF, and (**e**) 7AW/3KF.

The surface morphology results of fractured tensile samples for fibre biocomposites, including pure KF and AW, hybrid 3AW/7KF, 1AW/1KF, and 7KF/3AW, are shown here. These samples were tensile loaded, and the surface morphology from SEM is shown in Fig. 4a–e. During the matrix analysis, the fracture and expulsion of fibres were observed. Breakage of fibres occurred, indicating that interfacial adhesion between fibres and resin matrix was slightly stronger in hybrid samples than in pure AW and KF biocomposite. It is reasonable to believe that the stress can be successfully transferred from the matrix to the fibres. For pure samples, Fig. 4a,b indicated weak interfacial bonding between fillers and resin. Furthermore, the fracture surface showed omnidirectional rupture, which could be related to evidence of lower matrix strength below the stress level. The findings of the mechanical test were consistent with the SEM micrographs of biocomposites. These data indicate that the mechanical properties of AW/KF samples have improved. It is also clear from SEM micrographs that there is little evidence of fibre fracture in the composite, indicating that the hybrid biocomposites demonstrated good interfacial adhesion in addition to the resin matrix, resulting in improved effectiveness for stress transfer with improved mechanical characterisation when compared to the pure AW and KF biocomposites. Thus, the results of SEM indicated that the hybrid natural fibres could create the distinctive microstructure of this biocomposite, hence improving mechanical properties and promoting interfacial bonding between the fibres and epoxy matrix^53^.

### Flexural tests

**Fig. 5:**
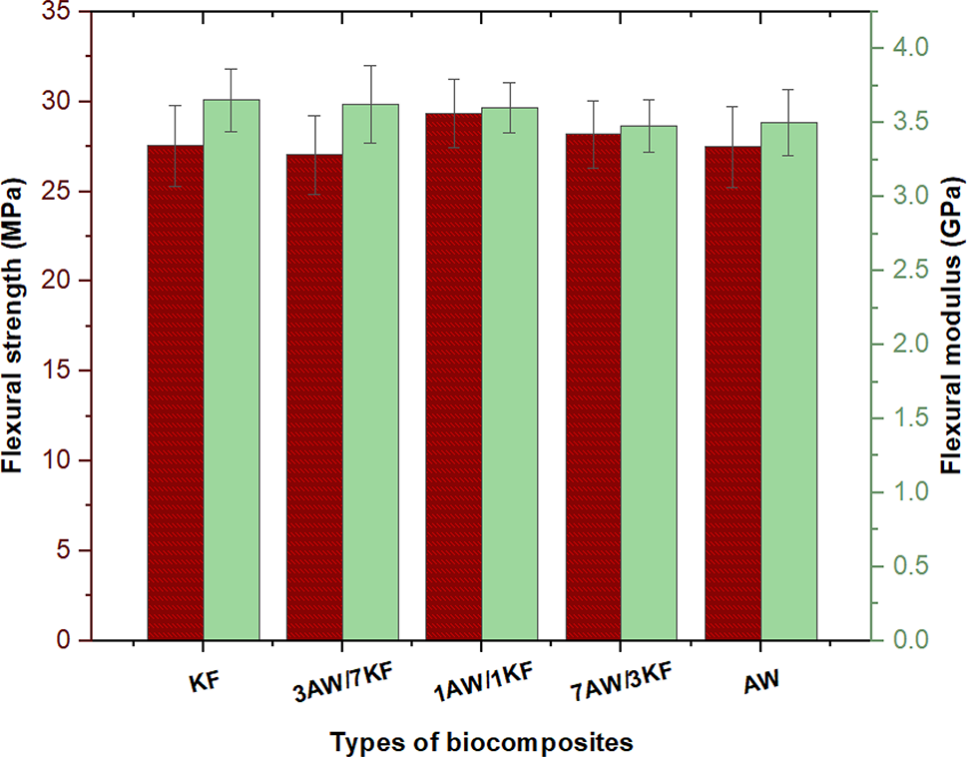
Flexural strength and moduli of pure and sample biocomposites.

Figure 5 depicts the flexural strength and modulus of pure and hybrid biocomposites, respectively. The minimum flexural strength of 3AW/7KF biocomposites was 27.056 MPa, while the highest flexural strength of 1AW/1KF biocomposites was 29.34 MPa, as shown in Fig. 5. The flexural strength of KF biocomposites, on the other hand, increased by about 0.2% when compared to AW biocomposites. In comparison, the flexural strength in the (7KF/3AW) sample was 28.18 MPa, indicating an improvement in flexural strength over the (3AW/7KF) sample. This could be owing to the hybridization of fibres, which results in strong bonding between matrix and fibre. This has resulted in great strength and stiffness of composites. As a result, the hybrid fibre incorporated with the resin has excellent adhesion capabilities, resulting in maximum flexural strength. Flexural modulus for KF biocomposites, on the other hand, increased by 2.82%, 4.89%, 1.39%, and 0.83% when compared to WA, 7AW/3KF, 1AW/1KF, and 3AW/7KF biocomposites, respectively. The hybrid biocomposites, on the other hand, showed a small drop in flexural strength. A related previous literature was performed by Albaqami et. Al. They observed that the highest flexural strength (78.90 MPa) was recorded in the KF hybrid biocomposites when compared to other biocomposites. As a result, the epoxy matrix may bond effectively to *Washingtonia* fibres, resulting in excellent flexural strength^54^. Gaagaia et al., investigated the mechanical properties of Washingtonia Filifera (WF) palm fibers (PF) hybrid biocomposites. They found that the tensile results, particularly, the young modulus of WF/PF increased up to 858.6 MPa, 17% elongation and a maximum stress of 15 MPa are found^55^.

### Impact properties

**Fig. 6:**
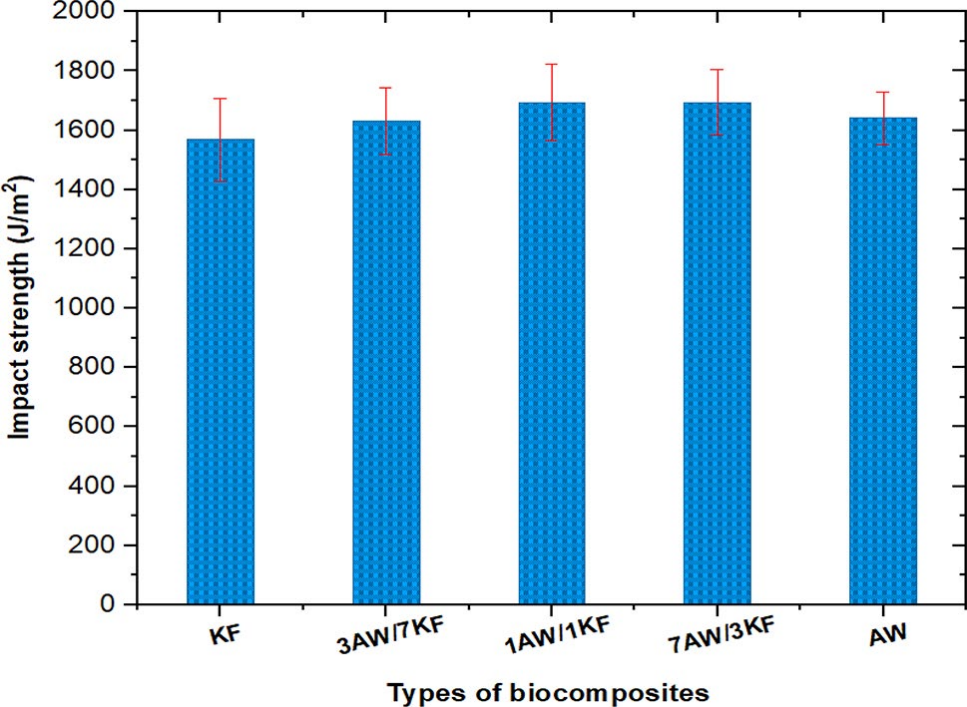
Impact strength of pure and sample biocomposites.

The impact strength of biocomposites appeared to be improved, which was attributed to the impact of hybridization and pure fibres reinforced with resin. The bonding strength between the fibres and the matrix is closely related to the impact properties of fibre-reinforced polymer composites. The impact strength of hybrid biocomposites was shown to be greater than that of pure biocomposites. Furthermore, as shown in Fig. 6, the impact strength of 1AW/1KF and 7AW/3KF biocomposites was identical, with a value of 1694 J/m^2^. The KF biocomposite, on the other hand, had the lowest value (1568 J/m^2^) while the AW pure biocomposite, the impact strength value was about 1640 J/m^2^, as shown in Fig. 6. Contrary, to other hybrid biocomposite (3AW/7KF), the impact strength value was 1630 J/m^2^. Several research works have been performed on the formulation of hybrid biocomposites and their performance has been examined^56^. It has been observed that hybrid biocomposites have demonstrated comparable strength to biocomposites with glass fibre reinforcement. Goud et al. improved roystonea/glass fibre-based epoxy hybrid composites. The hybrid composite revealed comparable impact strength (168 J/m) equally to that of the glass fibre epoxy biocomposite (169 J/m). Albaqami et al. studied the impact strength of KF/Jute hybrid composites, they found that the KF hybrid composites exhibited higher impact strength among all biocomposites while the increase in KF fibres in the matrix reduced the impact strength, which may be due to that further fibre subsequently the fibres contributed a little brittleness by increasing hardness, which caused in a drop in impact strength^54^.

**Fig. 7:**
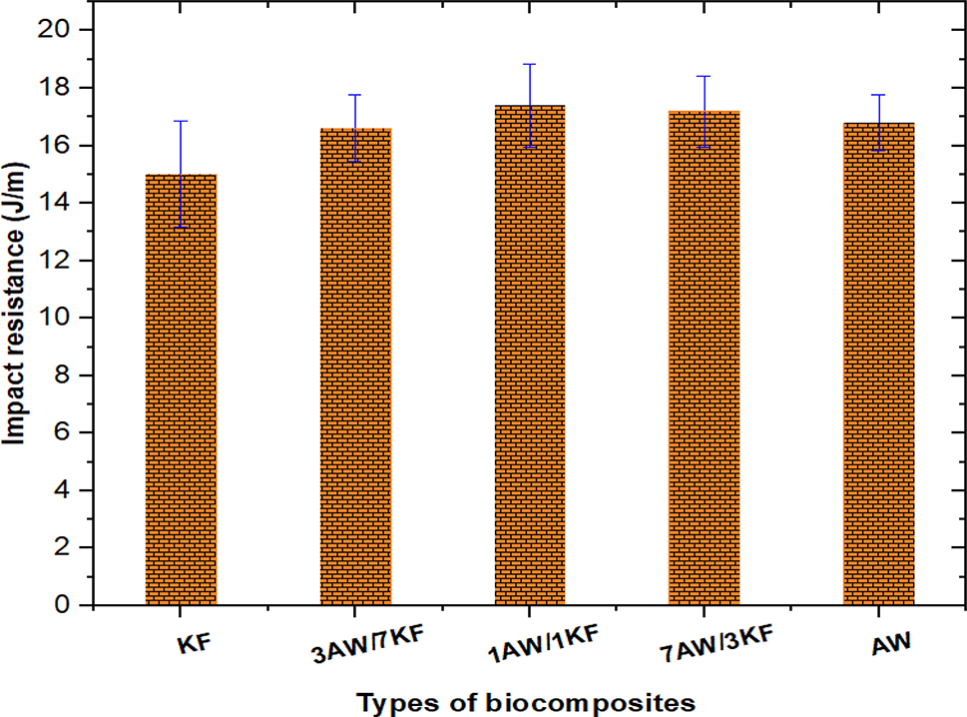
Impact resistance of pure and sample biocomposites.

The reason could be that the 1AW/1KF and 7AW/3KF biocomposites absorbed more energy as a result of good interlocking between the matrix and the fibre and better load transfer as a result of hyper dilations loading on the fibre surface, which prevented crack propagation during the impact test^54^. Figure 7 depicts the impact resistance of several types of fibre biocomposites. When compared to 7AW/3KF, 3AW/7KF, AW, and KF, the impact resistance of 1AW/1KF increased by 1.20%, 4.82%, 3.57%, and 16%, respectively, as shown in Fig. 7.

In comparison to related research works, Ismail et al. examined the hybrid biocomposites characterisation for samples fabricated from a kenaf (K) and bamboo (B) fibres epoxy biocomposite utilising the hand lay-up technique with 40 wt.% of fibre loading. Regarding this study, the 5B/5 K hybrid biocomposites showed the maximum impact strength, which was 45 J/m, contrasted to the pure kenaf bio composite and the 7B/3 K hybrid composites, which had impact strengths values of 40.8 and 37.9 J/m, respectively^57^

### Dimensional stability

**Fig. 8:**
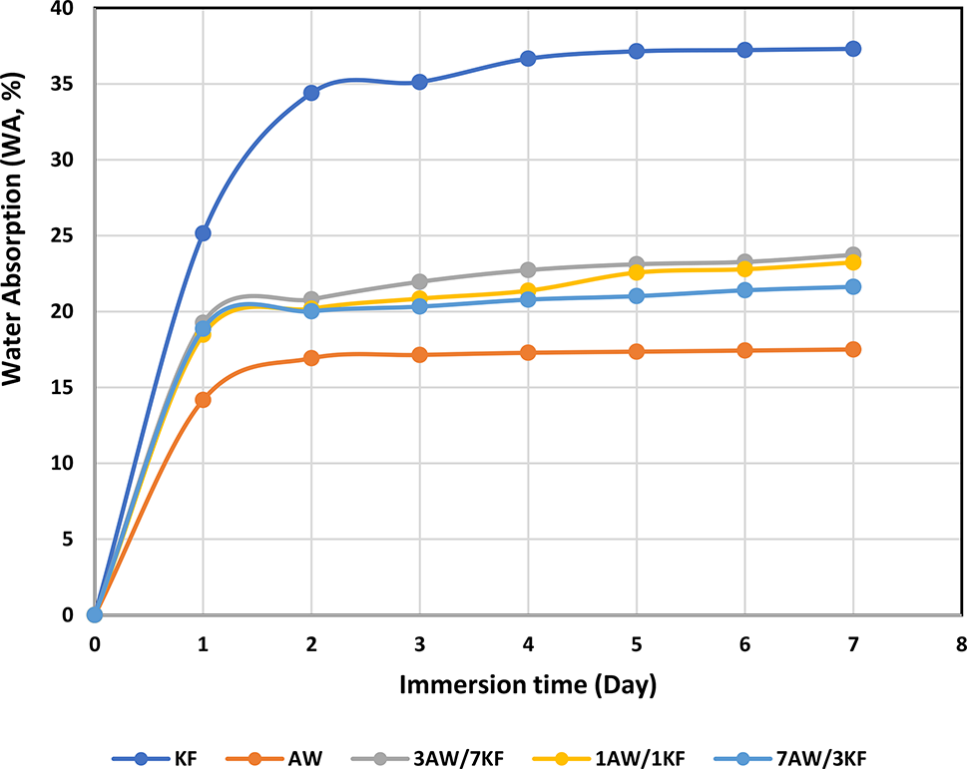
The WA (%) values of different biocomposite samples immersed at different times.

The relation between the water absorption (in %) values of biocomposites, and the duration of immersion is presented in Fig. 8. The introduction of pure biocomposite (KF) into the resin matrix increased the water absorption of biocomposites. The increase is slight rise with the maximum prolonged immersion time (7 days), with a maximum value of 37%, compared to the values of other samples (AW, 1AW/1KF, 3AW/7KF, and 7AW/3KF), which were 17.48%, 22.85%, 22.55%, and 21.65%, respectively, as shown in Fig. 8. On the other hand, the KF pure biocomposite exhibited the lowest WA value among all biocomposites, as shown in Fig. 8. This can be attributed, for instance, to poor interface adhesion between the KF and epoxy matrix^43^. The evaluation between hybrid biocomposites in Fig. 8 shows poor water absorption characteristics compared to pure KF and AW biocomposites. This might be attributed to comparatively existing hydroxyl (-OH) group reaction positions that promote the hydrophilic character in the pure biocomposites^58^. However, the lower water absorption of pure biocomposite might be attributed to some hydrophilic nature of pure natural fibres which have the cellulose and lignin-containing free hydroxyl group in their structure. Similar observations were made by Leman et. al, who claimed that the hydroxyl group of sugar palm can resist water due to lower cellulose content and lignin content, thereby giving lower absorption values^59^. Another study showed that the hybrid KF composites showed the lowest water absorption of 8% while the TS value was about 2%^60^.

**Fig. 9:**
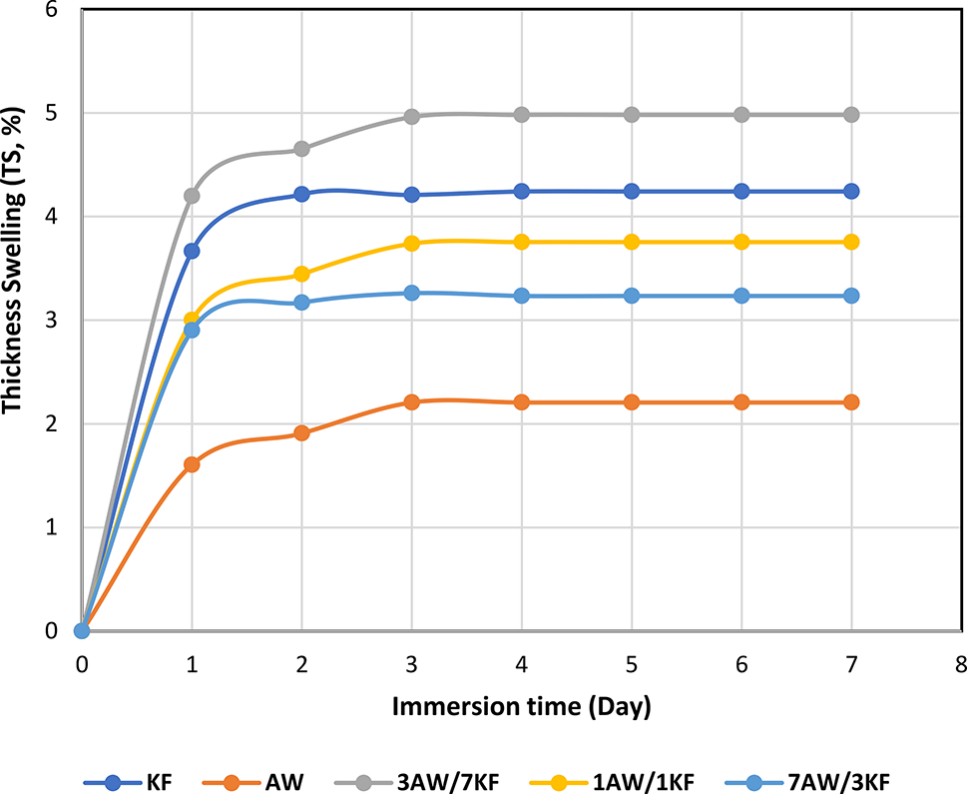
The TS (%) values of different biocomposite samples immersed at different times.

The biocomposite sample (3AW/7KF) was thicker on the 7th day exhibiting the greatest increases in thickness swelling (4.98%), while for AW, KF, 1AW/1KF, and 7AW/3KF biocomposites, the thickness swelling values increased by 2.20%, 4.24%, 3.75%, and 3.23%, respectively, as shown in Fig. 9. However, all samples exhibited more stability in the Ts values after 3 days of immersion. Furthermore, on the first day of immersion, 3AW/7KF biocomposites were observed to be 162%, 14.7%, 38.97%, and 44.72% thicker than the AW, KF, 1AW/1KF, and 7AW/3KF biocomposites. Regardless of the resin being nonpolar, it vaguely absorbs water, as revealed in Fig. 9. Hybrid biocomposites provided better interfacial adhesion between fibres and resin matrix, exhibiting fewer voids for water molecules to penetrate than pure biocomposites^61^.

These findings agreed with related previous research work by Razdi et. al. They investigated the behaviour of the TS and WA of hybrid biocomposites at different immersion times in distilled water. They noticed that the TS values were reduced for the roselle/sugar palm hybrid biocomposites compared to the pure samples. They suggested that with good interface and distribution between the two different fibres leads to reducing the swelling and percentage of dimensional stability for biocomposites^43^.

## Conclusions

A thorough methodology for the fabrication of bio-composites using various natural fibres was developed in this work. The tensile and flexural strength of the hybrid biocomposites increased, whereas elongation at break decreased. However, the tensile modulus of 7AW/3KF biocomposite was higher than that of other samples, while the flexural modulus of KF biocomposite was high. The hybrid biocomposite 7AW/3KF demonstrated the highest tensile strength at 16.05 MPa, significantly surpassing the pure KF and AW biocomposites, which had tensile strengths of 11.48 MPa and 11.87 MPa, respectively. Additionally, the AW biocomposite showed a higher tensile modulus of 3.3 GPa compared to KF’s 2.75 GPa, with a slight reduction in elongation at break from 0.59% to 0.48%. The AW pure biocomposite demonstrated a slightly higher impact strength of 1640 J/m^2^, while the 3AW/7KF hybrid biocomposite exhibited a comparable value of 1630 J/m^2^. These results suggest that certain hybrid configurations can enhance the impact strength of biocomposites compared to pure fibre composites. When exposed to the maximum time of immersion (7 days), the AW biocomposites had a lower water absorption capacity (17.5%) than the other biocomposites, whereas the 3AW/7KF biocomposite was thicker (4.98%) than the other biocomposites. Morphological investigation reveals that the interfacial adhesion between the fibres and the resin matrix enhanced in the hybrid biocomposites. These innovative hybrid biocomposites therefore can be used for various industrial applications in future, especially for automotive applications. The findings suggest that incorporating hybrid natural fibres can significantly enhance the mechanical performance of biocomposites, making them more suitable for various applications where higher strength and stiffness are required. Thus, they could be suitable for the packaging industry as biodegradability is the criteria for such applications.

## Data Availability

The datasets generated and/or analysed during the current study are not publicly available due to funded project, but are available from the corresponding author on reasonable request.
